# Exploring Vitamin D Deficiency and IGF Axis Dynamics in Colorectal Adenomas

**DOI:** 10.3390/biomedicines12081922

**Published:** 2024-08-22

**Authors:** George Ciulei, Olga Hilda Orășan, Angela Cozma, Vasile Negrean, Teodora Gabriela Alexescu, Simina Țărmure, Florin Eugen Casoinic, Roxana Liana Lucaciu, Adriana Corina Hangan, Lucia Maria Procopciuc

**Affiliations:** 1 4th Department of Internal Medicine, Faculty of Medicine, University of Medicine and Pharmacy “Iuliu Hațieganu”, 400012 Cluj-Napoca, Romania; george.ciulei@umfcluj.ro (G.C.); angelacozma@umfcluj.ro (A.C.); vasile.negrean@umfcluj.ro (V.N.); teodora.alexescu@umfcluj.ro (T.G.A.); simina.tarmure@umfcluj.ro (S.Ț.); eugen.casoinic@umfcluj.ro (F.E.C.); 2 Department of Pharmaceutical Biochemistry and Clinical Laboratory, Faculty of Pharmacy, University of Medicine and Pharmacy “Iuliu Hațieganu”, 400012 Cluj-Napoca, Romania; liana.lucaciu@umfcluj.ro; 3 Department of Inorganic Chemistry, Faculty of Pharmacy, University of Medicine and Pharmacy “Iuliu Hațieganu”, 400012 Cluj-Napoca, Romania; adriana.hangan@umfcluj.ro; 4 Department of Medical Biochemistry, Faculty of Medicine, University of Medicine and Pharmacy “Iuliu Hațieganu”, 400012 Cluj-Napoca, Romania; lprocopciuc@umfcluj.ro

**Keywords:** colorectal adenomas, vitamin D deficiency, insulin-like growth factor-1, insulin-like growth factor binding protein-3

## Abstract

(1) Colorectal cancer is a major cause of cancer-related death, with colorectal adenomas (CRAs) serving as precursors. Identifying risk factors such as vitamin D deficiency and the insulin-like growth factor (IGF) axis is crucial for prevention. (2) This case–control study included 85 participants (53 CRA patients and 32 controls) who underwent colonoscopy. We measured serum vitamin D3 (cholecalciferol), calcidiol (vitamin D metabolite), calcitriol (active vitamin D metabolite), insulin-like growth factor-1 (IGF-1), and insulin-like growth factor binding protein-3 (IGFBP-3) to explore their associations with CRA risk. (3) Results: We found that lower cholecalciferol levels were a significant risk factor for CRA (OR = 4.63, *p* = 0.004). Although no significant differences in calcidiol and calcitriol levels were observed between CRA patients and controls, calcidiol deficiency was common in the study population. IGF-1 levels inversely correlated with age, calcitriol, and IGFBP-3 in CRA patients. (4) This study highlights the potential of lower cholecalciferol levels to detect patients at risk of CRA when calcidiol values cannot, suggesting the importance of evaluating different vitamin D metabolites in cancer prevention research. Our findings underscore the need to further investigate the interactions between calcitriol, the active form of vitamin D, and the IGF axis in colorectal cancer development.

## 1. Introduction

Colorectal cancer (CRC) is one of the leading causes of cancer-related mortality worldwide, with colorectal adenomas (CRAs) being recognized as precursors to CRC. The identification and management of risk factors for CRA are crucial in the prevention of CRC. Among various factors (sedentary lifestyle, obesity, and Western diet), vitamin D has gained significant attention because of its potential role in cancer prevention [1]. Vitamin D (cholecalciferol), which is primarily obtained through sunlight exposure and diet, is metabolized in the liver to 25-hydroxyvitamin D (calcidiol), the main circulating form, and further converted in the kidneys to its active form, 1,25-dihydroxyvitamin D (calcitriol) [2]. Vitamin D deficiency, defined by serum calcidiol levels less than 20 ng/mL (50 nmol/L), is prevalent globally, with approximately 40% of Europeans reported to be deficient and 13% severely deficient. Severe deficiency, marked by calcidiol levels below 12 ng/mL (30 nmol/L), significantly increases the risk of various adverse health outcomes, including increased mortality and susceptibility to infections and chronic diseases [3].

The insulin-like growth factor (IGF) axis, particularly that of IGF-1 and its binding protein, insulin-like growth factor-binding protein-3 (IGFBP-3), is another area of interest in cancer research. There is an ongoing debate about whether reduced or elevated IGF-1 levels benefit or harm human health. People with genetic mutations in the IGF-1 pathway, such as those with Laron syndrome, have a reduced cancer risk, better insulin sensitivity, and lower diabetes risk [4], but may face increased stroke and cardiovascular disease risk [5]. Conversely, individuals with elevated growth hormone/IGF-1 signaling, such as those with acromegaly, have increased cardiovascular and cancer risks [6]. IGF-1 is thought to promote tumors by encouraging cell growth and preventing cell death. High circulating IGF-1 levels are associated with prostate and breast cancer [7], and some studies suggest associations with colorectal cancer [8].

A positive, bidirectional relationship has been observed between serum concentrations of IGF-1 and serum calcidiol levels. IGF-1 stimulates the production of 1-α-hydroxylase, promoting calcitriol synthesis in the kidneys. A high expression of vitamin D receptors (VDR) in liver and pituitary cells, which are central to IGF-1 and growth hormone secretion, can explain the influence of vitamin D metabolites on IGF-1 secretion [9]. The IGFBP-3 gene is a transcriptional target of VDR. Additionally, IGFBP-3 has been reported to inhibit VDR-dependent transcriptional activity [10].

This study aimed to investigate the prevalence of vitamin D deficiency in patients with CRA compared with controls and to explore the correlations between the serum levels of vitamin D metabolites (cholecalciferol, calcidiol, and calcitriol) and the IGF axis (IGF-1 and IGFBP-3). By examining these relationships, we seek to elucidate the potential role of vitamin D and the IGF axis as risk factors for CRA and CRC. Most research on vitamin D and its relationship with colorectal neoplasia has focused on calcidiol due to its longer half-life of approximately three weeks, making it a stable indicator of vitamin D status over time [11]. We decided to include serum measurements of cholecalciferol alongside calcidiol and calcitriol to provide a comprehensive assessment of vitamin D status. Measuring cholecalciferol allows us to evaluate the immediate availability of vitamin D before it undergoes hepatic conversion to calcidiol, thus reflecting recent vitamin D intake and synthesis more accurately. Additionally, cholecalciferol can diffuse directly into tissues and be locally converted to its active forms, suggesting its crucial role in maintaining local tissue-specific vitamin D levels [11].

## 2. Materials and Methods

### 2.1. Study Population

This case—control study involved a total of 85 participants, including 53 individuals diagnosed with colorectal adenoma (CRA) and 32 control subjects. All participants underwent colonoscopic examinations at the University Hospital C.F.R. in Cluj-Napoca, Romania, between February 2022 and December 2023. To be included in the case group, participants needed to be adults over the age of 30 years with a histopathologically confirmed diagnosis of CRA following their colonoscopy. The control group consisted of individuals who underwent colonoscopy for either routine CRC screening or due to symptoms suggestive of gastrointestinal issues (such as abdominal pain, changes in bowel habits, or rectal bleeding), that did not result in any pathological findings upon colonoscopy.

Participants were excluded from the study on the basis of several criteria to minimize variables that can influence the serum values of vitamin D metabolites, IGF1, and IGFBP-3. Individuals who refused to participate were excluded. Patients who were diagnosed with colorectal cancer or any other malignancy were not included. Participants with hereditary conditions such as familial adenomatous polyposis, Gardner syndrome, Lynch syndrome, or Peutz–Jeghers syndrome were excluded. Those with gastrointestinal conditions such as ulcerative colitis, Crohn’s disease, celiac disease, or a history of intestinal resection were also excluded to avoid confounding factors related to nutrient absorption and metabolism. Patients with liver cirrhosis and chronic kidney disease were excluded because of potential impacts on calcium and vitamin D metabolism. Participants who followed a diet that completely excluded dairy products were not included to avoid dietary inconsistencies that could affect the study results. Additionally, individuals who had supplemented with calcium or vitamin D in the previous six months were excluded to prevent recent supplementation from affecting baseline levels and study outcomes. Participants who were using antiepileptic drugs at the time were excluded because of their potential effects on vitamin D levels. Individuals with type I and II diabetes mellitus, acromegaly, or autoimmune diseases were excluded to maintain a homogeneous study population regarding metabolic and hormonal influences. Patients with conditions such as malnutrition, uncontrolled hypothyroidism or hyperthyroidism, heart failure, or active infections were excluded because of their potential to significantly impact overall health and study outcomes. Finally, pregnant women were also excluded.

Additionally, data on smoking history and family history were gathered. The colorectal adenomatous polyps identified during the study were categorized into various pathological types, including tubular adenoma, villous adenoma, tubulovillous adenoma, hyperplastic polyps, and serrated polyps. Vitamin D deficiency was defined as a serum value of calcidiol less than 20 pg/mL, and severe deficiency was defined as a value less than 12 pg/mL. Patients were classified as having high sun exposure if they were enrolled from April to September and as having low sun exposure if they were enrolled from October to March.

### 2.2. Blood Sample Collection and Laboratory Detection

Whole fasting blood samples were collected in EDTA tubes. The samples were then centrifuged promptly to separate the plasma, which was subsequently frozen and stored at −80 °C. The serum levels of IGF-1, IGFBP-3, cholecalciferol, calcidiol, and calcitriol were detected with the ELISA kits E-EL-H0086 (for IGF-1, Elabscience, Wuhan, China), E-EL-H0087 (for IGFBP-3, Elabscience, Wuhan, China), E0920Ge (for cholecalciferol, EIAab, Wuhan, China), DKO146 (for calcidiol, DiaMetra, Spello, Italy), and E0467Ge (for calcitriol, EIAab, Wuhan, China).

### 2.3. Statistics

Statistical analyses were performed via GraphPad Prism software version 9.0.0 (121). Quantitative continuous variables that adhered to a Gaussian distribution were summarized using the arithmetic mean and standard deviation (SD). For quantitative continuous variables that deviated from a Gaussian distribution, the median value and the interquartile range (IQR) (Q1 represents the 25th percentile, and Q3 represents the 75th percentile) were used. Qualitative nominal variables were described using both relative percentages and absolute frequencies (number of cases).

The serum levels of vitamin D metabolites (cholecalciferol, calcidiol, and calcitriol) and IGF-1 and IGFBP-3 were compared between CRA patients and control subjects. Correlations between these biomarkers, age, and BMI were assessed by Spearman’s correlation coefficients. The relationships between the serum concentrations of these markers and the risk of CRA were analyzed via logistic regression models, with ORs and 95% CIs calculated for different percentile levels (33% and 66%) of the biomarkers. Significant associations were further evaluated with multiple regression models to control potential confounders. To account for multiple testing, p values were corrected via the false discovery rate method, and an estimated Q value was calculated (significant if it was less than 0.10). Statistical significance was set at *p* < 0.05, and all tests were two-tailed.

## 3. Results

### 3.1. Demographic Data

Table 1 presents the demographic and clinical data for the patient groups, including those with CRA and those in the control group. The CRA group consisted of 53 patients, whereas the control group included 32 individuals. The average age of the CRA patients was 64.09 ± 10.18 years, which was significantly greater than the average age of the control patients (58.88 ± 10.67 years; *p* = 0.02). The sex distribution was similar across the groups, with 58% men and 42% women in the CRA group and 56% men and 44% women in the control group (*p* = 0.9). BMI and abdominal circumference were also measured, with no significant differences between the groups. With respect to lifestyle factors, 18% of the CRA patients and 28% of the controls reported a history of smoking (*p* = 0.61). Low sun exposure was reported by 43% of the CRA patients and 21% of the controls, with a trend toward significance (*p* = 0.06). In the CRA group, 45% of the patients had polyps larger than 1 cm, 84% had adenomatous polyps, 9% had serrated polyps, 15% had hyperplastic polyps, 30% had high-grade lesions, and 26% had multiple polyps.

### 3.2. Serum Values of the IGF Axis and Vitamin D Metabolites in the Study Groups

The vitamin D deficiency prevalence was 49% overall, and severe deficiency had a prevalence of 12%. In the CRA group, 26 subjects had vitamin D deficiency, 7 of whom had severe deficiency. Among those in the control group, 16 had vitamin D deficiency, and 4 had severe deficiency. Vitamin D deficiency was not associated with an increased risk of CRA (*p* = 0.9), nor was severe deficiency (*p* = 0.9).

Table 2 compares the serum values of cholecalciferol, calcidiol, calcitriol, IGF-1, and IGFBP-3 between CRA patients and control subjects, as well as between groups with low and high sun exposure. Across all measured biomarkers (cholecalciferol, calcidiol, calcitriol, IGF-1, and IGFBP-3), there were no statistically significant differences between CRA patients and controls or between the low- and high sun exposure groups.

### 3.3. Serum Correlations between IGF-1, IGFBP-3 and Vitamin D Metabolism

Table 3 presents the Spearman correlation coefficients and p values for the relationships between IGF-1 and IGFBP-3 with vitamin D metabolites, age, and BMI in the entire patient group, the CRA group, and the control group. In the entire patient group, IGF-1 was significantly positively correlated with IGFBP-3 (r = 0.25, *p* = 0.01) and significantly negatively correlated with cholecalciferol (r = −0.23, *p* = 0.03), calcitriol (r = −0.31, *p* = 0.003), and age (r = −0.40, *p* = 0.0001).

**Figure 1:**
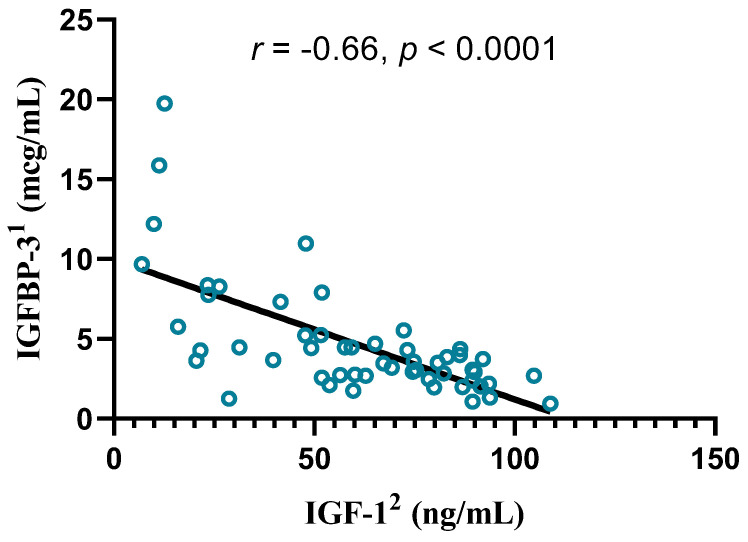
Correlation between IGF-1 and IGFBP-3 serum levels in the CRA^3^ group, Spearman’s r coefficient, *p* value, and a simple linear regression line. ^1^ IGFBP-3—insulin-like growth factor binding protein-3; ^2^ IGF-1—insulin-like growth factor-1; ^3^ CRA—colorectal adenoma.

In the CRA group, IGF-1 was significantly negatively correlated with IGFBP-3 (r = −0.66, *p* < 0.0001; Figure 1), calcitriol (r = −0.34, *p* = 0.01), and age (r = −0.39, *p* = 0.003).

**Figure 2:**
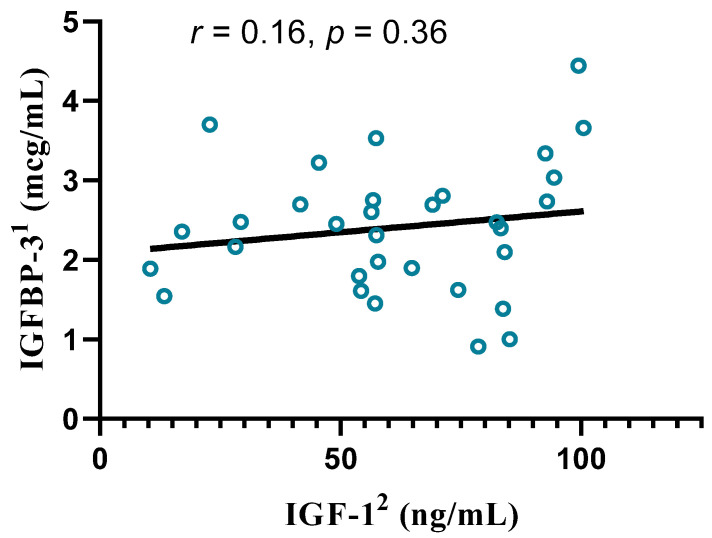
Correlation between IGF-1 and IGFBP-3 serum levels in the control group, Spearman’s r coefficient, *p* value, and a simple linear regression line. ^1^ IGFBP-3—insulin-like growth factor binding protein-3; ^2^ IGF-1—insulin-like growth factor-1.

In the control group, IGF-1 was significantly negatively correlated with age (r = −0.45, *p* = 0.01) but not with IGFBP-3 (Figure 2).

We also analyzed the correlations between the IGF1/IGFBP-3 ratio and parameters such as age and BMI. A negative correlation was found between age and the IGF1/IGFBP-3 ratio (r = −0.21, *p* = 0.04) for the entire population (r = 0.71, *p* < 0.001). When the CRA subgroup was separated from the control subgroup, only the control subgroup showed a negative correlation between the IGF1/IGFBP-3 ratio and age (r = −0.38, *p* = 0.03).

Regarding vitamin D metabolites, no significant correlation with age was observed for cholecalciferol (*p* = 0.61), calcidiol (*p* = 0.52), or calcitriol (*p* = 0.48) in the study population.

### 3.4. Serum Values of IGF-1, IGFBP-3 and Vitamin D Metabolites as Risk Factors for CRA

Table 4 provides the ORs and p values for the risk of CRA based on the percentile levels (33% and 66%) of vitamin D metabolites, IGF-1, and IGFBP-3. In the analysis, cholecalciferol levels ≤ 64.4 ng/mL were associated with a significantly increased risk of CRA (OR = 4.63, *p* = 0.004; adjusted OR = 3.858, *p* = 0.017, Q value = 0.07). Cholecalciferol levels ≥ 78.34 ng/mL were not significantly associated with CRA risk (OR = 0.68, *p* = 0.47). No associations were found between different percentile levels of calcidiol, calcitriol, IGF-1, and IGFBP-3.

## 4. Discussion

Our study highlighted several findings regarding CRA patients compared with our control group. First, the demographic data indicated that the average age of the CRA patients was significantly greater than that of the control patients. Other factors, such as BMI and smoking history, did not significantly differ between our two groups. Advancing age is known to increase the risk of CRA, supporting the recommendation for timely screening to enhance the early detection and prevention of CRC [12].

Our analysis revealed that there were no significant differences in the serum levels of vitamin D metabolites between the two groups. The prevalence of vitamin D deficiency in our subjects was similar to that reported in European populations [3]. In our study, lower levels of calcidiol, which is the metabolite typically used to assess vitamin D status, were not a risk factor for CRA. Our results contrast with those of others, who reported that calcidiol deficiency is associated with an increased risk of CRA and CRC [13]. In other studies, only hyperplastic polyps were more prevalent in vitamin D-deficient subjects [14]. In a pooled analysis of 2074 patients, low calcidiol values were significantly inversely associated with the presence of three or more adenomas at baseline and with villous histology. Low calcidiol levels are also significantly associated with the development of three or more recurrent lesions [15]. In a meta-analysis of 15 studies, a significant inverse relationship was found between circulating calcidiol levels and CRA (OR = 0.68; 95% CI: 0.54–0.82) [16]. Because of these results, the next question to be asked is whether vitamin D supplementation can prevent CRA or CRC. In the VITAL trial, which involved 25,871 participants free of cancer and cardiovascular disease at enrollment, daily vitamin D supplementation (2000 IU) did not reduce the risk of CRA [17]. The difficulty in conducting vitamin D supplementation studies consists of the variability of the dosages used (less than or more than 2000 UI/day), the timing of treatment (daily or intermittent), the duration of treatment, and the addition of calcium intake. Even so, vitamin D supplementation has been found to lower all-cancer mortality in randomized controlled trials [18].

A significant result of our study was the presence of lower cholcelciferol values as a risk factor for CRA, since studies on vitamin D usually do not measure this parameter. Cholecalciferol is considered by some to be better than calcidiol in determining vitamin D deficiency. Cholecalciferol has a half-life of approximately one day, whereas calcidiol has a half-life of several weeks. This means that serum cholecalciferol can quickly reflect recent intake and sun exposure, whereas calcidiol levels are more stable and do not show short-term changes [11]. Many cells possess the enzymes needed to convert cholecalciferol to calcidiol (CYP2R1 in the liver, as expected, but also in human brain pericytes, adipose tissue, or prostate) or vitamin D to calcitriol (CYP27B1 in bone cells, melanocytes, activated macrophages, keratinocytes, and the brain and endocrine glands) [11,19]. Cholecalciferol, especially in its free form, is more likely to diffuse into cells and be converted locally to its active form. The binding dynamics and greater free fraction of cholecalciferol than of calcidiol support its direct cellular availability and relevance. Historically, humans have continuously synthesized vitamin D from constant sun exposure. The body’s mechanisms are adapted to steady vitamin D levels rather than the fluctuations seen with intermittent dosing, making daily serum cholecalciferol levels a more natural and accurate measure [11]. Follow-up research should investigate the effectiveness of different forms of vitamin D supplementation in preventing CRC. Given our findings that lower cholecalciferol levels are a risk factor for CRA, it is essential to compare daily dosing with a weekly dosing of vitamin D. This comparison is important because weekly dosing may not maintain consistently high cholecalciferol levels, even if calcidiol levels appear adequate. Another important aspect to investigate is the impact of vitamin D supplementation on VDR gene expression at the tissue level in CRA or CRC. Although it has been shown that vitamin D supplementation can influence VDR expression [20], further research is needed to understand how this applies specifically to adenoma or CRC tissue. This investigation should also consider different VDR genotypes, as they influence CRC risk [21].

We did not find a significant association between the serum levels of vitamin D metabolites and the time of year of blood draw. This result has also been observed in other studies, likely due to the prevalence of a lifestyle that favors more indoor activities [13].

The inverse relationship between IGF-1 and age is known [22] and was also found in our cohort. Studies have reported elevated serum levels of IGF-1 in patients with CRA. Patients with polyps located in the proximal colon presented significantly higher IGF-1 serum concentrations than did those with distal colon polyps. Increased IGF-1 serum levels are also associated with a heightened risk of advanced adenomas [23]. Despite this, Renehan et al. reported that IGF-1 serum levels were similar between patients with small adenomas and those without adenomas. They also reported that IGF-1 serum levels were greater in patients with advanced adenomas than in individuals without adenomas [24]. These results were not observed in our cohort or in other studies [25,26].

We observed a negative correlation between IGF-1 and IGFBP-3 in the CRA group. In the control group, these parameters were not correlated. The role of IGFBP-3 in colonic carcinogenesis has been inconsistent across studies. Schoen et al. reported higher serum IGFBP-3 concentrations in patients with advanced colorectal adenomas than in controls [27]. Conversely, several studies reported no significant differences in IGFBP-3 levels between adenoma patients and non-adenoma patients, irrespective of adenoma advancement [26,28]. Serum IGF-1 values have been reported to be inversely correlated with BMI, but the bioavailability of IGF-1, measured by the ratio of IGF-1/IGFBP-3, can increase with BMI [29]. In our study, the ratio was inversely related to age in controls but not in patients with CRA, suggesting greater bioavailability for IGF-1 in patients diagnosed with CRA. This result is in support of the greater activity of the IGF-axis as a risk factor for CRA.

We found a negative correlation between IGF-1 and calcitriol in the CRA group but not in the control group. In the study by Trummer et al., a randomized controlled trial that measured the effects of vitamin D supplementation on IGF-1 in hypertensive patients, calcitriol correlated positively with IGF-1 both before and after the intervention [30]. The relationship between vitamin D and IGF-1 has been examined across various demographics. Ameri et al. reported a positive correlation between vitamin D levels and IGF-1 in adults with growth hormone deficiency, observing significant increases in circulating IGF-1 with increased vitamin D supplementation [31]. A randomized controlled trial in healthy children indicated that higher doses of vitamin D supplementation led to elevated serum IGF-1 levels [32]. A meta-analysis revealed a nonsignificant increase in IGF-1 following vitamin D supplementation overall. However, doses of less than 1000 IU/day and intervention durations of less than 12 weeks were associated with significant increases in IGF-1 levels, whereas higher doses and longer durations led to significant decreases. Initially, this relationship appears to be positive and dose-dependent; however, beyond a certain threshold of vitamin D supplementation, a negative feedback mechanism may reduce IGF-1 levels [33]. This can in part explain the negative correlation that we observed. Considering the correlations that we identified, future studies should investigate how serum levels of calcitriol and other vitamin D metabolites influence tissue expression of IGF-1 and IGFBP-3 in CRA or various stages of CRC development.

Our study has certain limitations. The lower sample size of our study limited the extent of the subgroup analysis we could perform. This constraint also impacts the extrapolation of our findings to broader populations. As such, caution must be exercised when generalizing our results beyond the studied cohort. Future research with larger sample sizes is necessary to confirm these findings and enhance their applicability to more diverse groups. Since vitamin D and calcium are metabolically interconnected, it is crucial to evaluate whether the relationship between calcidiol levels and CRA differs on the basis of calcium intake. Unfortunately, this study did not have data on individual calcium intake. Additionally, we did not measure general sun exposure for each of the subjects. One other limitation of the study was that it did not measure insulin levels or IGF-1 serum levels. Since insulin and IGF-1 are closely linked in regulating growth and metabolism, by not including insulin measurements, we might be missing important information about how these hormones together influence the development of CRA. Many studies on vitamin D and health outcomes consider the role of metabolic syndrome. By not including these components, our study may be less comparable to the literature, limiting our ability to validate or challenge previous findings. The strengths of our study lie in the analysis of multiple vitamin D metabolites in relation to the IGF-axis in this particular group of patients. Most studies have only determined calcidiol in patients with CRC or CRA without comparing the values of cholecalciferol or calcitriol.

## 5. Conclusions

The findings of this study highlight several critical aspects of the relationships among vitamin D metabolites, the IGF axis, and CRA. Although no significant differences in the serum levels of calcidiol and calcitriol were observed between CRA patients and controls, lower cholecalciferol levels were identified as a significant risk factor for CRA. This underscores the potential importance of evaluating cholecalciferol, rather than solely focusing on calcidiol, in assessing vitamin D status and its association with CRA risk. The study also revealed significant inverse correlations between IGF-1 levels and age, IGFBP-3 level and calcitriol level in CRA patients, suggesting that the IGF axis might play a role in CRA development.

## Figures and Tables

**Table 1:** Demographic and clinical data in patient groups.

	CRA ^1^ Patients (n = 53)	Controls (n = 32)	*p*
Age	64.09 ± 10.18	58.88 ± 10.67	0.02
Gender	31 men (58%) 22 women (42%)	18 men (56%) 14 women (44%)	0.9
BMI ^2^	29.0 ± 4.7	29.3 ± 4.70	0.8
Abdominal circumference	103.2 ± 12.6	102.8 ± 13.1	0.86
Smoking history	12 (18%)	9 (28%)	0.61
Low sun exposure	23 (43%)	7 (21%)	0.06
Polyp > 1 cm	24 (45%)		
Adenomatous polyps	45 (84%)		
Serrated polyps	5 (9%)		
Hyperplastic polyps	8 (15%)		
High-grade lesions	16 (30%)		
Multiple polyps	14 (26%)		

^1^ CRA—colorectal adenoma; ^2^ BMI—body mass index.

**Table 2:** Serum values of cholecalciferol, calcidiol, calcitriol, IGF-1 ^1^, IGFBP-3 ^2^ in patient groups.

	CRA ^3^ Patients (n = 53)	Controls (n = 32)	*p*	Low Sun Exposure Group (n = 23)	High Sun Exposure Group (n = 62)	*p*
Cholecalciferol (ng/mL)	70.9 ± 14.9	74.0 ± 11.1	0.3	72.2 ± 15.0	72.0 ± 12.9	0.96
Calcidiol (pg/mL)	20.2 (15.0325.35)	19.3 (13.3–27.5)	0.72	19.6 ± 7.1	20.1 (15.0–28.9)	0.38
Calcitriol (pg/mL)	34.9 (29.1–41.3)	37.9 (30.0–45.8)	0.26	35.3 (28.7–42.2)	30.7 (29.5–44.3)	0.21
IGF-1 (ng/mL)	62.7 (40.6–84.7)	61.4 ± 25.8	0.9	59.0 ± 24.9	66.0 (37.5–84.4)	0.5
IGFBP-3 (mcg/mL)	2.1 ± 0.9	2.4 ± 0.7	0.17	2.3 ± 0.8	2.1 ± 0.8	0.36

^1^ IGF-1—insulin-like growth factor-1; ^2^ IGFBP-3—insulin-like growth factor binding protein-3; ^3^ CRA—colorectal adenoma.

**Table 3:** Correlation coefficient (Spearman’s r) and *p* values for IGF-1 ^1^ and IGFBP-3 ^2^ with vitamin D metabolites in the CRA ^3^ group and control group.

Entire Patient Group	IGF-1 and IGFBP-3	IGF-1 and Cholecalciferol	IGF-1 and Calcidiol	IGF-1 and Calcitriol	IGF-1 and Age	IGF-1 and BMI ^4^
Spearman’s *r* coefficient	0.25	−0.23	0.05	−0.31	−0.40	−0.05
*p*	0.01	0.03	0.63	0.003	0.0001	0.61
Entire patient group		IGFBP-3 and cholecalciferol	IGFBP-3 and calcidiol	IGFBP-3 and calcitriol	IGFBP-3 and age	IGFBP-3 and BMI
Spearman’s *r* coefficient		−0.07	0.01	−0.09	−0.20	0.06
*p*		0.50	0.92	0.36	0.06	0.53
CRA group	IGF-1 and IGFBP-3	IGF-1 and cholecalciferol	IGF-1 and calcidiol	IGF-1 and calcitriol	IGF-1 and age	IGF-1 and BMI
Spearman’s *r* coefficient	−0.66	−0.24	−0.12	−0.34	−0.39	0.05
*p*	<0.0001	0.07	0.37	0.01	0.003	0.70
CRA group		IGFBP-3 and cholecalciferol	IGFBP-3 and calcidiol	IGFBP-3 and calcitriol	IGFBP-3 and age	IGFBP-3 and BMI
Spearman’s *r* coefficient		0.10	0.01	0.22	0.15	0.19
*p*		0.46	0.94	0.10	0.25	0.15
Control group	IGF-1 and IGFBP-3	IGF-1 and cholecalciferol	IGF-1 and calcidiol	IGF-1 and calcitriol	IGF-1 and age	IGF-1 and BMI
Spearman’s *r* coefficient	0.16	−0.22	0.28	−0.28	−0.45	−0.29
*p*	0.36	0.22	0.11	0.10	0.01	0.10
Control group		IGFBP-3 and cholecalciferol	IGFBP-3 and calcidiol	IGFBP-3 and calcitriol	IGFBP-3 and age	IGFBP-3 and BMI
Spearman’s *r* coefficient		−0.11	−0.11	−0.23	−0.05	−0.31
*p*		0.52	0.52	0.19	0.76	0.07

^1^ IGF-1—insulin-like growth factor-1; ^2^ IGFBP-3—insulin-like growth factor binding protein-3; ^3^ CRA—colorectal adenoma, ^4^ BMI—body mass index.

**Table 4:** Risk of CRA ^1^ based on percentile levels (33% and 66%) of vitamin D metabolites, IGF-1 ^2^, and IGFBP-3 ^3^.

	OR (95% CI) ^4^	*p*	Adjusted OR (95% CI) ^(a)^	*p* ^(a)^	Q-Value ^(b)^
Cholecalciferol ≤ 64.4 ng/mL	4.63 (1.67–12.7)	0.004	3.858 (1.33–12.68)	0.017	0.07
Cholecalciferol ≥ 78.34 ng/mL	0.68 (0.28–1.71)	0.47	1.53 (0.58–4.08)	0.38	
Calcidiol ≤ 16.23 pg/mL	0.63 (0.26–1.54)	0.35	0.67 (0.25–1.79)	0.78	
Calcidiol ≥ 22.94 pg/mL	0.68 (0.28–1.71)	0.47	1.49 (0.56–4.01)	0.41	
Calcitriol ≤ 31.87 pg/mL	1.54 (0.62–3.81)	0.47	1.23 (0.46–3.43)	0.67	
Calcitriol ≥ 40.64 pg/mL	0.68 (0.28–1.71)	0.47	1.57 (0.59–4.20)	0.35	
IGF-1 ≤ 51.9 ng/mL	1.54 (0.62–3.81)	0.47	0.92 (0.31–2.76)	0.89	
IGF-1 ≥ 78.62 ng/mL	0.87 (0.36–2.27)	0.81	0.90 (0.31–2.49)	0.84	
IGFBP-3 ≤ 1.82 mcg/mL	1.9 (0.78–5.28)	0.23	1.43 (0.51–4.16)	0.49	
IGFBP-3 ≥ 2.64 mcg/mL	0.72 (0.29–1.81)	0.63	1.32 (0.50–3.45)	0.56	

^1^ CRA—colorectal adenoma; ^2^ IGF-1—insulin-like growth factor-1; ^3^ IGFBP-3—insulin-like growth factor binding protein-3; ^4^ OR—odds ratio. ^(a)^ ORs and *p* values adjusted for age, sex and smoking status; ^(b)^ Q-value < 0.10 using the False Discovery Rate adjustment for multiple testing.

## Data Availability

The raw data used in this study can be obtained upon reasonable request to Lucia M. Procopciuc (luciamariaprocopciuc@yahoo.com) and George Ciulei (george.ciulei@umfcluj.ro).
